# Efficacy of Botox versus Placebo for Treatment of Patients with Major Depression

**Published:** 2017-07

**Authors:** Abbas ZAMANIAN, Atefeh GHANBARI JOLFAEI, Golnaz MEHRAN, Zahra AZIZIAN

**Affiliations:** 1. Rasoul-e-Akram Hospital, Iran University of Medical Sciences, Tehran, Iran; 2. Skin and Stem Cell Research Center, Tehran University of Medical Sciences, Tehran, Iran; 3. Dept. of Psychiatry, Iran University of Medical Sciences, Tehran, Iran

**Keywords:** Botox, Treatment, Major depression

## Abstract

**Background:**

Treatment of major depression is crucial to decrease the burden of disease. Hence, in this study, the efficacy of Botox was compared with placebo for treatment of patients with major depression.

**Methods:**

In this randomized clinical trial, 28 consecutive patients with major depression were enrolled. The patients were randomly assigned to receive either Botox or placebo and the scores of Beck Depression Inventory were determined and compared at baseline and after two and six weeks in the groups and between the groups. In addition, the drug adverse effects were compared between groups. This study had been registered in TCTR with TCTR20170409001 code.

**Results:**

There was a statistically significant difference two group for 6^th^ week Beck Score (*P*=0.004), but at baseline and after two weeks, there was no significant difference (*P*>0.05). None of the patients experienced side effects.

**Conclusion:**

Finally, Botox is effective for treatment of patients with major depression and it has a high safety.

## Introduction

Depression is a common psychiatric disorder leading to high burden especially for some other psychiatric comorbidity (1). Annually 43.7 billion dollars are expended for patients with depression among them 28% are directly for depression and other costs are related to mortality and morbidity due to depression (2). However, routine depression treatments include tricyclic antidepressants and selective serotonin reuptake inhibitors (2, 3); use of other therapeutic methods such as complementary treatments (3) or injection methods such as botulinum toxin (Botox) are suggested in some studies (4, 5).

Botox is a drug with multiple applications in dermatology treatments especially with cosmetic purposes (6). Among main applications, treatment and reduction of facial wrinkles may be mentioned (7–9). However, different variables including physician’s skill, used dose, therapeutic goals, hypersensitivity history, and causes of Botox injection are contributing to outcomes and adverse effects (6). However, high safety and good efficacy of this drug are represented (10), there are scarce studies among Iranian patients.

In this study, the efficacy of Botox was compared with placebo for treatment of patients with major depression.

## Materials and Methods

In this randomized clinical trial done in Rasoul-e-Akram Hospital, Tehran, Iran in 2014–2015, 28 consecutive patients with major depression (according to DSM-V) and Beck Depression Inventory were enrolled. The inclusion criteria were lack of hypersensitivity to Botox, and satisfaction for enrollment in the study and the exclusion criteria were lack of possibility for follow-up and lack of satisfaction for incorporation in the study. The main anti-depressant treatments were alike between groups and no alteration was done in routine treatments.

Local Ethical Committee approved the study and Helsinki Declaration was respected all over the study course.

Incorporating subjects signed the informed consent form. The patients were randomly assigned to receive either Botox or placebo and the scores of Beck Depression Inventory were determined and compared at baseline and after two and six weeks in the groups and between the groups. In addition, the drug adverse effects were compared between groups.

Data analysis was performed among 28 subjects including 14 subjects in control group and 14 patients in case group. Data analysis was performed by SPSS ver. 13.0 (Chicago, IL, USA) software. Chi-Square, Fisher, and Independent-Sample-*t* tests were used and were considered statistically significant at *P*-values less than 0.05.

## Results

The mean age was 35.14±11.8 and 43.71±10.9 yr in Botox and placebo group, respectively. In each group, 7 subjects were male and 7 patients were female. In each group, 71.4% had normal body mass index, 14.3% were overweight, and 14.3% were obese. Six subjects (42.9%) in Botox and 11 patients (78.6%) in placebo group were married. Nine subjects (64.3%) and seven patients (50%) in Botox and placebo groups were smoker. Five subjects (35.7%) and eight patients (57.1%) in Botox and placebo groups had history of alcohol consumption. Six subjects (42.9%) and five patients (35.7%) in Botox and placebo groups had positive family history of major depression. The mean duration of disease was 3.71 ± 2.3 and 3.50 ± 2.4 yr in Botox and placebo group, respectively. There was a statistically significant difference two group for 6^th^ week Beck Score, but at baseline and after two weeks, there was no significant difference (Table 1). None of the patients experienced side effects.

**Table 1:** Beck scores in two groups across the study

**Time**	**Botox Group**	**Placebo Group**	***P* Value**
Baseline	30.86 ± 5.35	27.71 ± 3.75	0.083
Two Weeks	27.29 ± 6.14	25.07 ± 4.16	0.274
Six Weeks	19.00 ± 4.82	24.29 ± 4.04	0.004

## Discussion

In this study, the efficacy of Botox was compared with placebo for treatment of patients with major depression and encouraging effects were seen after six weeks of treatment. However, there was no significant difference after two weeks. Despite good efficacy of Botox glabellar injection, further studies should be performed to obtain more definite results about efficacy and safety of these methods for treatment of major depression (11). This matter shows the importance of our study for approving the efficacy of Botox.

There are some controversies about efficacy of Botox in treatment of major depression and further studies such as our study are required (12). Overall, 47 percent efficacy for Botox versus 9% for placebo that is in congruence with our findings (13).

The decrease in facial sadness by Botox was reported as well as our study. However, they had no control comparison group (14). Decreased depression was reported in patients with blepharospasm under treatment with Botox that is in congruence with our findings (15). A study (16) among ten patients with major depression revealed improvement in nine subjects showing similar results with our study.

## Conclusion

Botox is effective for treatment of patients with major depression and it has a high safety. However, further studies with larger sample size and multi-center samplings are required to attain results that are more definite.

## Ethical considerations

Ethical issues (Including plagiarism, informed consent, misconduct, data fabrication and/or falsification, double publication and/or submission, redundancy, etc.) have been completely observed by the authors.
